# Quantitative Image Analysis of Epithelial and Stromal Area in Histological Sections of Colorectal Cancer: An Emerging Diagnostic Tool

**DOI:** 10.1155/2015/569071

**Published:** 2015-10-22

**Authors:** R. Rogojanu, T. Thalhammer, U. Thiem, A. Heindl, I. Mesteri, A. Seewald, W. Jäger, C. Smochina, I. Ellinger, G. Bises

**Affiliations:** ^1^Department of Pathophysiology and Allergy Research, Medical University of Vienna, 1090 Vienna, Austria; ^2^TissueGnostics GmbH, 1020 Vienna, Austria; ^3^Clinical Institute of Pathology, Medical University of Vienna, 1090 Vienna, Austria; ^4^Seewald Solutions, 4616 Weisskirchen/Traun, Austria; ^5^Department of Clinical Pharmacy and Diagnostics, University of Vienna, 1090 Vienna, Austria; ^6^Faculty of Automatic Control and Computer Engineering, “Gheorghe Asachi” Technical University of Iasi, 700050 Iasi, Romania

## Abstract

In colorectal cancer (CRC), an increase in the stromal (S) area with the reduction of the epithelial (E) parts has been suggested as an indication of tumor progression. Therefore, an automated image method capable of discriminating E and S areas would allow an improved diagnosis. Immunofluorescence staining was performed on paraffin-embedded sections from colorectal tumors (16 samples from patients with liver metastasis and 18 without). Noncancerous tumor adjacent mucosa (*n* = 5) and normal mucosa (*n* = 4) were taken as controls. Epithelial cells were identified by an anti-keratin 8 (K8) antibody. Large tissue areas (5–63 mm^2^/slide) including tumor center, tumor front, and adjacent mucosa were scanned using an automated microscopy system (TissueFAXS). With our newly developed algorithms, we showed that there is more K8-immunoreactive E in the tumor center than in tumor adjacent and normal mucosa. Comparing patients with and without metastasis, the E/S ratio decreased by 20% in the tumor center and by 40% at tumor front in metastatic samples. The reduction of E might be due to a more aggressive phenotype in metastasis patients. The novel software allowed a detailed morphometric analysis of cancer tissue compartments as tools for objective quantitative measurements, reduced analysis time, and increased reproducibility of the data.

## 1. Introduction

Colorectal cancer (CRC) is a heterogeneous and multifactorial disease like other solid tumors [1, 2]. This might explain why tumors identical in the morphology have different responses to therapeutic interventions leading to alterations in the survival rates [3–5]. For a more precise classification of the tumors as well as a better estimation of the prognosis, potent molecular and/or protein markers are clearly needed in addition to the currently used histological grading (G) and* Tumor-Node-Metastasis (TNM) staging system*.

More and more, the importance of the stroma (S) surrounding the epithelial cells (E) is recognized [6]. Many histological studies on S as the tumor microenvironment have been undertaken to investigate the cellular heterogeneity in CRC [7]. Thereby, the interplay between E and S structures such as matrix components and immune cells has been investigated [8]. For example, tumor growth was found to be influenced depending on the subset of tumor infiltrating lymphocytes [9] and presence of tumor associated macrophages was associated with an improved overall survival [10]. More widespread venous and lymphatic invasion is a prognostic parameter for metastases [11]. Finally, an increased area of S within the tumor and a decrease in the E/S ratio predicts poor survival in CRC [12, 13], as well as in breast [14], and in esophageal cancer patients [15]. The E/S ratio was therefore suggested to be included in the individual risk estimation [12]. So far, all data on E/S were determined manually (semiquantitative) on Haematoxylin and Eosin (H&E) stained sections in the most invasive tumor area by 2-3 experts [13–15].

The precision of analysis was improved by morphometric methods, for example, point counting on H&E stained tumor sections with a virtual Graticule software, where objects were included in categories like tumor, stroma, vessel, inflammation, and so forth [12].

However, the reproducibility of the visual (human-based) quantitative evaluation is time consuming and difficult to achieve, while quick qualitative assessment (scoring) may be more error-prone and with higher interobserver variability.

To overcome these problems, the aim of this study was to establish a method for the automated analysis of E and S in large tumor sections. To generate a fast and reproducible quantification of E/S, we performed immunofluorescence staining studies using the keratin 8 marker for E in paraffin-embedded sections from CRC patients with and without liver metastasis. We developed a novel automated microscopic image analysis method based on segmentation for E and S quantification and used the immunofluorescence (IF) technique to stain the tissue. This would allow quantification of additional markers in relation to epithelial compartment by a multistaining IF process, such as proximity to tumor or ratio of expression inside epithelium versus stroma.

## 2. Material and Methods

### 2.1. Patients

We studied formalin-fixed and paraffin-embedded tumor samples from CRC patients with (*n* = 12) and without (*n* = 14) liver metastasis undergoing surgical curative resection at the Department of Surgery at the General Hospital of Vienna between 1995 and 2007. None of the patients received preoperative chemo- or radiotherapy. All patients had grade 2 (G2) tumors, belonging to a large heterogeneous group of patients with respect to the other grades, which would benefit from better prognostic and predictive factors [3]. The clinicopathological characteristics of the patients are shown in Table 1.

As controls, colon mucosa samples distant from the cancerous areas were used (*n* = 5). Normal colon tissue samples were taken from patients undergoing gastric bypass surgery (*n* = 4). Informed consent was obtained from all patients and the study was approved by the Ethics Committee of the Medical University of Vienna (protocol number 358/2010).

### 2.2. Immunofluorescence Staining

Following deparaffinization and rehydration, tissue sections (4 *μ*m) were boiled for 20 minutes in 0.05% citraconic anhydride for antigen retrieval.

After permeabilization with phosphate buffered saline (PBS)/0.2% Tween and blocking with 5% goat serum (Dianova GmbH/Jackson ImmunoResearch, USA) in 0.05% Tween/PBS, sections were incubated with rabbit anti-human keratin 8 antibody (clone EP1628Y, Thermo Fisher Scientific, Fremont, CA, USA) for 1 hour followed by incubation with DyLight 549 goat anti-rabbit (Vector Laboratories, Burlingame, CA, USA) for 1 hour. To verify validity of K8 as marker for epithelial cells which might acquire mesenchymal properties, *β*-catenin double immunolabelling was performed. For double staining, after permeabilization and blocking (as described above), sections were incubated with rabbit anti-human *β*-catenin (Abcam, Cambridge, UK) overnight at 4°C followed by incubation with DyLight 549 goat anti-rabbit IgG (Vector Laboratories, Burlingame, CA, USA). For the second staining, sections were incubated with mouse anti-human keratin 8 antibody (Clone TS1, Thermo Fischer Scientific, Fremont, CA, USA) for 1 hour and as a secondary antibody Alexa Fluor 647 goat anti-mouse IgG (Invitrogen Molecular Probes/Life Technologies, Paisley, UK) was used. 4′,6-Diamino-2-phenylindole (Dapi; Roche Diagnostics GmbH, Vienna, Austria) was applied at 0.2 *μ*g/mL in PBS for 10 min to visualize cell nuclei. The negative control was prepared by either omitting or replacing the primary antibodies by nonimmune rabbit and mouse IgG, respectively. After washing, the slides were mounted with Fluoromount G (SouthernBiotech, Birmingham, AL, USA).

All samples were processed under standardized conditions to minimize results variations due to the technical procedure. For automated tissue segmentation by EPSTRA, epithelial cells were detected by using only the single immunolabelling of keratin 8 (K8).

If not stated otherwise, chemicals were obtained from Sigma-Aldrich (St. Louis, MO, USA).

### 2.3. Acquisition with TissueFAXS

Tissue sections were acquired using a TissueFAXS plus tissue cytometer (TissueGnostics GmbH, Vienna, Austria) using the 20x magnification in fluorescence scanning mode. For segmentation purposes (entire tissue, epithelial area, and lumen), fluorescence intensity information from 4 different fluorescence channels (DAPI, FITC/Cy2, mCherry/TxRed, and Cy5) was collected, regardless, whether a specific staining was present or not (see also Region-Based Segmentation Approach). In the double immunolabelling experiments, signals from the Cy5 channel (K8), mCherry/TxRed (*β*-catenin), and DAPI (nuclei) were collected. The DAPI staining pattern of nuclei allows the estimation about the quality of the tissue and the efficacy of the staining procedure in individual samples. The following filter sets (Chroma 49000 series filter sets) were used: DAPI (350 nm excitation, 400 nm dichroic, and 460 nm emission), FITC/Cy2 (470 nm excitation, 495 nm dichroic, and 525 nm emission), mCherry/TxRed (560 nm excitation, 585 nm dichroic, and 630 nm emission), and Cy5 (620 nm excitation, 660 nm dichroic, and 700 nm emission). Previews of the entire slides were taken using a 2.5x objective on the DAPI channel. On these images, the tissue sections were outlined for scanning with a 20x high power magnification objective. The same autofocus settings were used for all slides, enabling one autofocus point for each 3 × 3 group of fields of view. To ensure full contrast conditions, the integrated extended focus feature was applied by setting five different z-levels with a 2 *μ*m interval (2 above and 2 below the z-level detected by autofocus). The z-stack images were merged into one critically sharp image. Fluorescence images were acquired and stored with lossy compression in JPEG format, 95% quality index. For each Field of View (FOV), individual monochrome images were captured separately for all selected pixel shift-free filter sets. For presentation, false colors replaced the monochrome image.

### 2.4. Definition of the Regions of Interest (ROIs)

The median scanned area per slide was 205 mm^2^ (ranging from 64 to 315 mm^2^). The area included in individual ROIs (defined by the user) was 1–26 mm^2^ depending on the structure of the section.

On the whole slide overview of the image section, ROIs were drawn manually using the zooming and mark-up tools included in the StrataQuest 5.0 software, part of TissueFAXS plus solution. The need to analyze and compare different regions within the tumor tissue was derived from the observation that the proliferation compartment [16] as well as the expression of proteins like *β*-catenin and cyclin D1 [17] was not equally distributed in the tumor mass. Heterogeneities in the structures were also seen in noncancerous parts of the mucosa in CRC samples [18].

**Figure 1:**
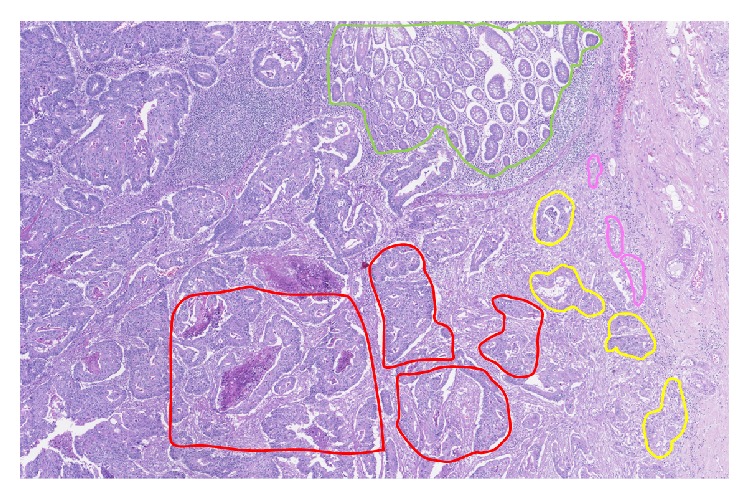
Hematoxylin and Eosin stained section of a CRC tissue with regions drawn as follows: adjacent mucosa region is framed in green, tumor center in red, invasive front 1 in yellow, and invasive front 2 in pink.

To better illustrate the selection of the regions based on their characteristic appearance, a Hematoxylin and Eosin stained section of a CRC tissue was divided into different areas (Figure 1).

**Figure 2:**
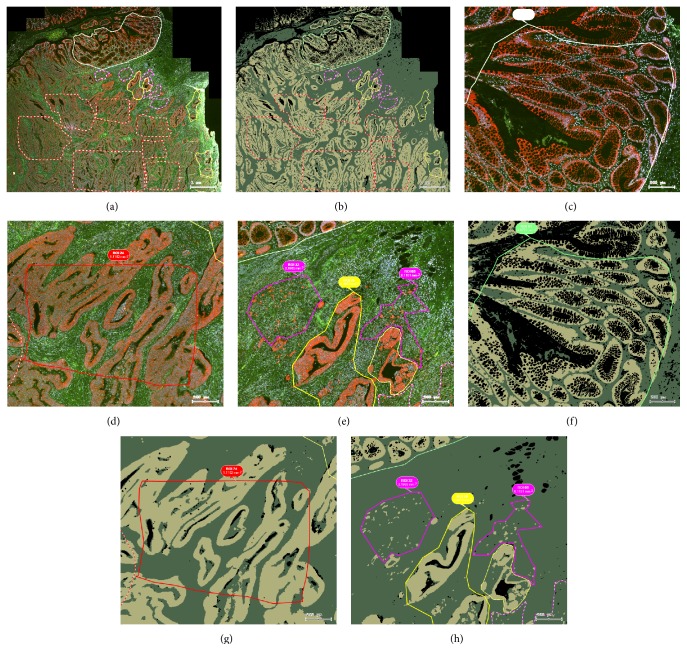
Example of an image of a CRC tissue section analyzed by EPSTRA. (a) K8 staining (TxRed) overlaid with DAPI (blue) including Cy2 (green) and Cy5 (white) background fluorescence; (b) epithelial mask (yellow) and stromal mask (green) as found by EPSTRA; (c) the adjacent mucosa is depicted in green contours, (d) tumor center in red, (e) invasive front 1 in yellow, and invasive front 2 in pink; (f)–(h) show the corresponding epithelial mask of (c)–(e) views. Bubbles show the region of interest (ROI) identification number and its area. Bar indicates 200 *μ*m.

Under supervision of an experienced pathologist (I. Mesteri) the areas are categorized as follows:

- Normal mucosa: from healthy patients (scanned area: 5–9 mm^2^).
- Normal-appearing mucosa (scanned area: 4–12 mm^2^): distant from the cancerous areas in CRC samples.
- Adjacent mucosa: the normal-appearing mucosa adjacent to the tumor (depicted in green in Figures 1, 2(a), 2(b), and 2(c), scanned area: 1–26 mm^2^).
- Tumor center: central part of the tumors with dense glandular structures surrounded by S, located >0.5 cm away from the tumor border (depicted in red in Figures 1, 2(a), 2(b), and 2(d), scanned area: 1–24 mm^2^).
- Invasive front 1: the invasive margin of the tumor area where the glandular structures reach out into the submucosal S but still form a compact mass (depicted in yellow in Figures 1, 2(a), 2(b), and 2(e), scanned area: 1–8 mm^2^).
- Invasive front 2: the tumor area facing the submucosal S with infiltrating E structures composed of single K8 positive cells or small K8 positive cell clumps (up to 10 cells) (depicted in dark pink in Figures 1, 2(a), 2(b), and 2(e), scanned area: 0–2 mm^2^).

The regions were drawn around the epithelial cells located on the border of the tumour area to the stroma to avoid any inclusion of area which contains exclusively stroma. Artifacts, such as folded or broken tissue areas or areas showing strong background staining due to autofluorescence of erythrocytes and lipofuscin, were excluded from the analysis.

### 2.5. New Algorithms

The most important and difficult step in automatic image analysis is the image segmentation, which provides critical information for further image understanding. The aim of the new segmentation algorithms is to highlight the E and S areas as well as the lumen for a proper computation of the E/S ratio. The outlining should be accurate enough even in tumor areas that have thin S compartments. By analyzing the content of scanned images, the following challenges were noticed:

- Different grey values for the positive areas (nonuniform staining).
- Some cell clusters containing very few cells (they may represent tumor buds).
- Holes in the K8 grayscale channel due to lack of K8 antigen inside the nuclear compartment.
- Some nonspecific areas appearing more intensely stained than the positive K8 (erythrocytes, auto-fluorescence, lipofuscin, folded tissue, nonuniform tissue reactivity, etc.).
- Multiple intensity-classes of pixels, each displaying multiple peaks in the histogram: lumen, S, weak E, intense E, folded S, folded E, and erythrocytes.
- Erythrocytes being intense in both TxRed and GFP channels.
- E areas which can be very close to each other. In the center of the tumor, the S may only form a very narrow band between crypts (as thin as 5 pixels wide).
- Big data (up to 100.000 images per slide, each image with 1.4 mega-pixels).

The characteristics and novelty of the proposed method are given by the way of integrating the particularity of these biological signals (the four input channels and the intensities distribution for E, S, and lumen) into widely accepted fast image preprocessing and segmentation methods.

### 2.6. Region-Based Segmentation Approach

Since our goal was to find the boundaries for the areas of interest we used region-based segmentation based on Otsu's thresholding method [19]. Let one consider that *T*_ref_ = thr_Otsu_(*I*) is the threshold computed with this method on the entire input image *I*. Segmenting the entire image with the same global threshold (*T*_ref_) could cause the loss of some epithelial or stroma areas due to intrastaining variability. Therefore, we used an adaptive threshold applied on image tiles (*T*_*i*_) as provided by the TissueFAXS file format.

A disadvantage of Otsu thresholding is that it separates the image in two pixel classes even though the image may have only one class of pixels (e.g., stroma could be detected within a tile only with lumen). In addition, it may fail when provided with images with a multitude of the classes present in the images (e.g., lumen, weak stroma, intense stroma, and epithelium). Thus, we implemented a restriction such that the threshold is accepted only if it is within a predefined interval: thr_Otsu_(*T*_*i*_) ∈ [*𝒯*_ref_ − *α*, *𝒯*_ref_ + *α*], where *α* is a constant. We chose the reference threshold as the one given by Otsu thresholding applied on the entire virtual slide.

If the obtained threshold is outside the accepted restricted interval, the process is repeated by considering only the pixels within the predefined interval [*𝒯*_min_, *𝒯*_max_] empirically established after analyzing multiple image sets. All the pixels with the intensity smaller than *𝒯*_min_ and all the pixels with the intensity higher than *𝒯*_max_ are eliminated. Applying this transformation before the Otsu thresholding guaranties the proper segmentation of the two classes of interest. We named this approach “Adaptive Guided Segmentation”:

(1) $$\begin{array}{c} \text{AGS}\left(\;I,T_{min},T_{max}\;\right). \end{array}$$

Since the acquired images present artifacts, a simple yet efficient enhancement technique was used: after the background is removed by subtracting an appropriate predefined constant (*c*), the intense areas are further emphasized by raising values of the pixels to a certain power (*p* > 1) empirically determined for each channel:

(2) $$\begin{array}{c} Ie={\left(\;I-c\;\right)}^{p}. \end{array}$$

None of the required regions (lumen, stroma, and epithelial region) can be accurately extracted from a single channel due to aforementioned intensity variability. Therefore, a combination of these channels was used to create more robust “virtual channels” (VC). Thus, a mix of enhanced DAPI, TxRed, FITC, and Cy5 was made for tissue detection (VC_tissue_) and one for epithelial detection (VC_epi_):

(3) VCtissue=IeDAPI+IeGFP+IeTxRed+IeCy5,VCepi=IeTxRed−IeGFP−IeCy5.

The algorithm workflow needed two branches: one for lumen (L_mask_) versus tissue (T_mask_) masks identification and one for stroma (S_mask_) versus epithelial (E_mask_) identification within the tissue mask (T_mask_). The tissue-lumen detection phase extracts the tissue and lumen masks, respectively, by applying AGS on VC_tissue_:

(4) $$\begin{array}{c} \left\{\;L_{mask},T_{mask}\;\right\}=AGS\left(\;{VC}_{tissue},T_{min}^{1},T_{max}^{1}\;\right). \end{array}$$

Subsequently, the epithelial and stroma masks were extracted using AGS on the VC_epi_ only within tissue mask T_mask_:

(5) $$\begin{array}{c} \left\{\;S_{mask},E_{mask}\;\right\}=AGS\left(\;{VC}_{epith}{,T}_{min}^{2},T_{max}^{2}\;\right)⋂T_{mask}. \end{array}$$

AGS generates irregular contours typical to binarization methods. Therefore, we smoothed the contours by applying a series of closing and opening morphological operators [20] further referred to as AGSm. Since larger smoothing filters may generate a better smoothing at the cost of losing details, we preferred using filters not larger than 5 pixels.

**Figure 3:**
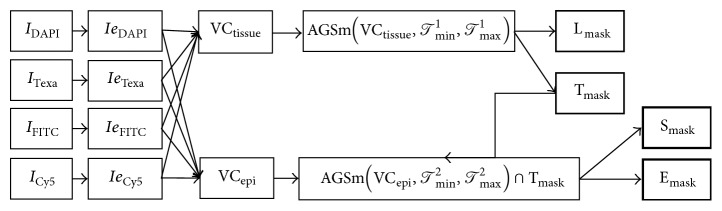
Processing data flow.

The final data flow of the implemented segmentation technique is presented in Figure 3.

Due to the huge amount of data which needed to be processed, the proposed algorithm/segmentation technique was implemented using a parallel approach making use of the independent computing units (cores) available on current processors. On a 16-core computer, an improvement of 14-fold in the analysis speed was noticed compared to sequential single-core approach, reaching an average time of 3 min per section.

### 2.7. Measurements and Statistical Processing

Masks of E and S areas were saved as images corresponding to original image tiles. StrataQuest 5.0 was used to create analysis projects from the original images, as well as from the newly created mask files. With the module “Total Area Measurement,” the calculation was done for the areas of two masks, for every single marked group of regions: normal mucosa, mucosa, adjacent mucosa, tumor center, invasive front 1, and invasive front 2.

Batch export functionality extracted the final measurements in a single Excel file containing measurements of area of E and S as well as their ratio, for each group of region on each slide. Finally, the remaining statistics (averages, standard deviations) were calculated in Excel.

## 3. Results

### 3.1. K8 as a Marker for Epithelial Cells in CRC

**Figure 4:**
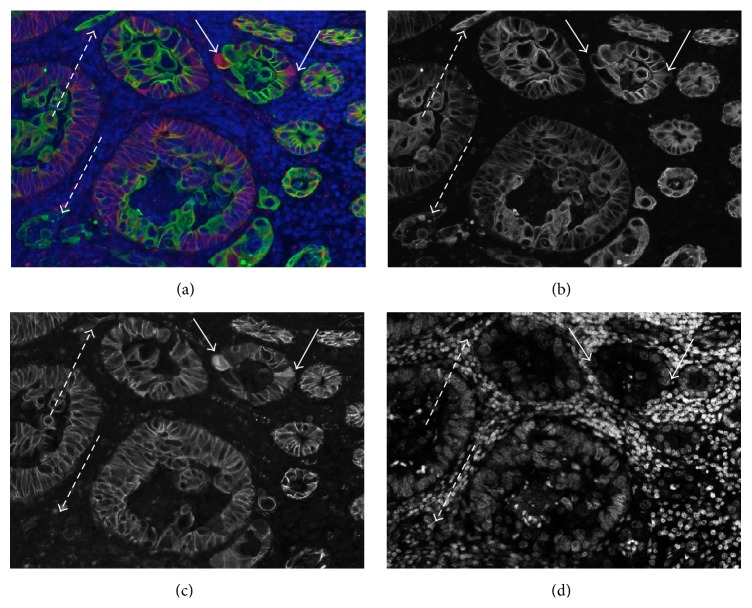
*β*-catenin/K8 double staining in a colorectal cancer tissue section. (a) Merged image: *β*-catenin positive cells are visible in red, and K8 positive cells are shown in green. Nuclei are shown in light blue (all false colors). The same image is shown with individual fluorescence channels in grey scale: (b) K8, (c) *β*-catenin, and (d) nuclear staining with DAPI. Arrows with solid lines indicate cells with a translocation (cytoplasmic or nuclear) of *β*-catenin. Arrows with dashed lines indicate small groups of cells still positive for K8, but no more expressing *β*-catenin.

To differentiate between E and S area in CRC sections, we used the epithelial cell marker K8 [21]. To verify that the expression of K8 is appropriate to label epithelial cells at various cancer stages in all patient groups, we performed on an additional labeling with *β*-catenin on 5 test slides which were checked visually. In normal epithelial cells, *β*-catenin is located on the cellular membrane at the lateral sides of the cells [22]. During cancer progression, *β*-catenin accumulates in the cytoplasm and hence enters the nucleus (see examples in Figures 4(a) and 4(c)).

However, the expression of *β*-catenin is lost in some epithelial cells within the tumor (Figures 4(a) and 4(c), arrows with dashed lines), but a strong expression of K8 is maintained in all epithelial cells within all areas. Thus, we conclude that K8 can be considered a reliable marker for normal as well as for cancerous epithelial cells in the colon.

### 3.2. Region Definition and Assessment of EPSTRA Mask Results

StrataQuest can overlay any combination of channels and masks in various colors and transparencies.  Figure 2 shows examples of such overlays in the way they were used for defining and categorizing ROIs. EPSTRA mask and contour results could be also overlaid for visual assessment.

### 3.3. Comparison of EPSTRA versus Results from Human Experts and Other Automated Methods

Evaluation of the method was done on a test dataset consisting of 24 FOVs from all defined compartments and patient groups. When assessing the capability of algorithms, the ground truth is considered to be results of human experts [23]. To compensate for interobserver variability, we considered the opinion of three different human experts. By including them separately in the performance assessment, we can compare the results from each individual against the performance of the proposed EPSTRA method. The ground truth (GrTr) was created by averaging the manual mark-up (average GrTr), which assigned pixels from the entire test dataset to three masks: lumen, S, and E. After eliminating the lumen mask, pixels classified by the method and by the averaged GrTras E were considered as “true positives” (TP). Also, we calculated “false positive” (FP = pixels marked as A by the algorithm but not by the GrTr), “false negative” (FN = pixels not marked as E by the algorithm but marked by the GrTr), and “true negative” (TN = pixels not marked as E by neither the algorithm nor by the GrTr). Precision, recall/sensitivity, specificity, and balanced F1 score [24] were calculated as follows:

(6) Specificity=TNTP+FP,Precision=TPTP+FP,Recall=TPTP+FN,F1=2∗Precision∗RecallPrecision+Recall.

As alternative automated workflows, we implemented Otsu global thresholding (GT) and local thresholding variant by processing each image tile independently (LT). Both these two binarization methods were enhanced with additional morphological postprocessing (MP) to eliminate the holes situated in the place of the nuclei (methods named GT + MP and LT + MP, resp.). We also evaluated the newer method shown in [25] using morphological hierarchy (MH), although it was designed for and tested on healthy tissue only. Considering the results of enumerated alternative methods, we calculated the specificity, precision, recall, and F1 score. Data is summarized in Table 2.

### 3.4. Evaluation of the E/S Ratio in the Defined Compartments

**Figure 5:**
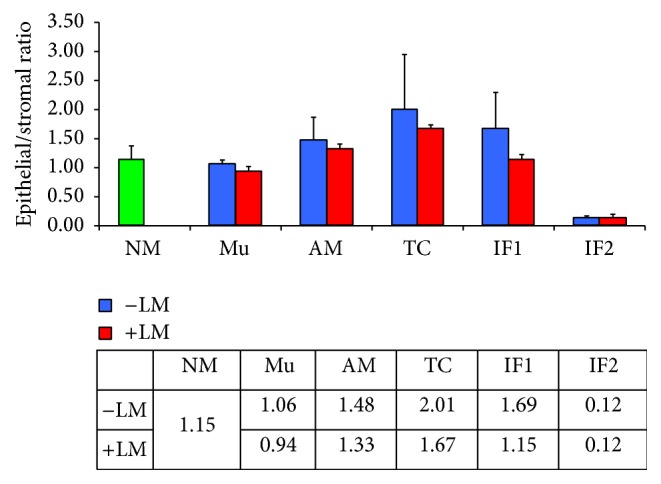
Epithelial/stromal ratio in different tissue compartments (normal mucosa (NM) in healthy patients, normal appearing mucosa (Mu) distant from tumor, adjacent mucosa (AM), tumor center (TC), invasive front 1 (IF1), and invasive front 2 (IF2)) in colorectal cancer patients with and without liver metastasis. Values are given as means ± standard deviation.

Data on the epithelial/stromal ratio generated by EPSTRA is summarized in Figure 5.

In healthy colon sections, the mean ratio of 1.15 for normal mucosa (NM) indicates that the amount of E roughly equals the amount of S. This ratio is similar in the normal appearing mucosa (Mu) distant from tumor area in CRC sections (0.94 for patients with and 1.06 for patients without liver metastasis). In the mucosa adjacent (AM) to the tumor, the mean ratio is slightly higher (1.33 for patients with and 1.48 for patients without liver metastasis). An even higher amount of E area was found within the tumor center with an E/S of 1.67 for patients with and 2.01 for patients without liver metastasis. At the invasive front 1 (IF1), the E/S in patients without liver metastasis was 1.69 and it was reduced in that with liver metastasis (1.15). As expected, at the invasive front 2 (IF2), where the tissue is disrupted and only single tumor cells or small clamps of cells are spread in the stroma, the E/S ratio is very low (mean ratio of 0.12 for patients with and without liver metastasis, resp.).

## 4. Discussion

We established an automatic microscopic imaging method to measure the E and S area in paraffin-embedded section from CRC patients. The novel algorithms for the calculation of the E/S ratio offer significant improvements of the histological evaluation of immunofluorescent stained tumor sections. In comparison with the H&E staining, identification of epithelial cells is increased due to specific labelling of keratin 8. Virtual microscopy overlaying techniques bring additional advantages when defining regions of interest based on both morphology and intensity of the stained markers.

Image processing techniques are used mainly because they allow large scale statistical evaluation in addition to classical eye screening. As noticed in Table 2, the new EPSTRA methods achieved better results than existing automatic approaches, almost reaching the performance of human experts. The “GT” method succeeds in extracting more relevant threshold values but fails in adapting to existing local variations of the sample (i.e., tumor center shows lower overall expression levels). On the other side, “LT” does not find proper values in areas without lumen or stroma. EPSTRA uses information from GT but then continues to adapt to local variations succeeding in proper segmentation despite intrinsic expression variability. In addition, the fact that it could find both thin S areas inside tumor centers and small E structures (which may represent tumor buds) embedded in the S made it superior when compared to all other evaluated automated methods. In addition, it performed at least as good as the worse human expert delineating contours in a digital slide with a digital pen at original resolution. Since scoring (quick visual qualitative assessment) is typically faster and more error-prone than manual drawing of contours, our automated method would perform better than such visual scoring procedures. Morphological postprocessing typically helps to remove very small holes or structures (up to 5 pixels). However, using MP for holes/structures bigger than 5–10 pixels dramatically decreases contour accuracy and the recognition rate of thin S and/or isolated E cells. These problems are also specific to MH [25] and will also appear in other approaches that are based on image downscaling. However, EPSTRA successfully avoided these shortcomings by working with images at original resolution (pixel size = 0.323 *μ*m).

Using EPSTRA on the entire image dataset, we found an increase in E in the central part of the tumor, as it was expected from the uncontrolled growth of E cells. At the tumor margins (IF1), the E/S ratio in CRC patients with liver metastasis is 32% lower than in patients without liver metastasis. These findings suggest that the lower amount of E at the tumor margin is related to metastasis and could be caused by epithelial-mesenchymal transition (EMT) of tumor cells. A similar reduction of E/S ratio was noticed in TC as well. Thus, patients with the reduced E/S ratio may have a shorter progression-free survival time as suggested from previous visual assessment studies in colon and breast cancer [12–14].

In tumor with increases in S areas, E clusters seem to be less compact. The invasive margin shows a rupture of the E structure and even budding events. Single E cells or small E cell clusters are floating in the S. This histomorphological structure [26] at the tumor margin is already proposed as a parameter to be included in predicting CRC prognosis [27]. Since budding is difficult to assess visually, the newly designed EPSTRA method proves to be adequate for quantification of this surrogate marker. Although the method requires more extensive immunostaining and sophisticated imaging than traditional visual histopathology, it offers important benefits. It could provide more additional information than simply counting the tumor buds. Furthermore, EPSTRA could allow multiple biomarkers to be quantified selectively over specified cell types, regardless of their abundance.

## 5. Conclusion

In this study we developed the EPSTRA method for automated compartment based morphometric analysis and determination of the E/S ratios in CRC sections as a means of advanced tissue cytometry. We demonstrated that EPSTRA can be used as a powerful tool for objective quantitative measurements of tumor structural development, with a scalable analysis time and reproducibility of data. In the future, applying this technique and elaborating more elaborated scoring procedures together with additional IF markers may extend diagnostic options leading to a fast and reliable characterization of tumor properties in individual patients. Thereby it may help enabling a better personalized treatment and individual follow-up in the patient's evaluation, promoting an improvement of the efficacy of a certain therapeutic regimen [28].

## Figures and Tables

**Table 1:** Clinicopathological characteristics of the study population.

	−liver metastasis	+liver metastasis
*Number of patients*	14	12
*Age, median (range)*	68 (53–78)	68 (35–79)
*Male (%)*	12 (86%)	8 (67%)
*TNM classification*		
pT		
pT1-2	6	0
pT3-4	8	12
pN^*∗*^		
pN0	13	6
pN1-2	1	6
*Dukes classification*		
A	4	0
B	8	6
C	1	5
D	0	1
Unknown	1	0
*Location*		
Coecum	3	5
Colon ascendens/transversum/descendens	1/3/1	0/1/0
Sigma/rectum	2/2	2/3
Unknown	2	1

^*∗*^No patients have more than 2 lymph node metastases.

**Table 2:** Specificity, precision, recall, and F1 score from three human experts (hExp 1–3), alternative automated methods (GT, GT + MP, LT, LT + MP, and MH), and EPSTRA.

Method/expert	Specificity	Precision	Recall	F1 score
hExp1	0.97	0.99	0.98	0.98
hExp2	0.98	0.99	0.97	0.98
hExp3	0.98	0.99	0.98	0.99
GT	0.97	0.99	0.34	0.51
GT + MP	0.98	0.99	0.30	0.47
LT	0.93	0.98	0.75	0.85
LT + MP	0.96	0.99	0.74	0.85
MH	0.96	0.99	0.80	0.89
**EPSTRA**	**0.97**	**0.99**	**0.97**	**0.98**

GT: global thresholding using cumulative histogram thresholding; MP: morphologic processing; LT: local thresholding; MH: morphological hierarchy.
